# Dectin-1 and dectin-2 drive protection against *Sporothrix brasiliensis* in experimental sporotrichosis

**DOI:** 10.3389/fimmu.2025.1668445

**Published:** 2025-09-25

**Authors:** Fabio Seiti Yamada Yoshikawa, Sandro Rogerio de Almeida, Shinobu Saijo

**Affiliations:** ^1^ Division of Molecular Immunology, Medical Mycology Research Center, Chiba University, Chiba, Japan; ^2^ School of Pharmaceutical Sciences, Department of Clinical e Toxicological Analysis, University of São Paulo, São Paulo, Brazil

**Keywords:** *Sporothrix brasiliensis*, Dectin-1, Dectin-2, IL-17, Treg

## Abstract

**Introduction:**

The emerging fungal pathogen *Sporothrix brasiliensis* has been responsible for epidemic outbursts of sporotrichosis in Latin America, particularly Brazil, in recent years. The higher aggressiveness of the infection and its zoonotic nature are hallmarks of the pathogen, but the immunological markers of protection are not fully characterized. The C-type lectin receptors – dectin-1 and dectin-2 – drive key antifungal responses, and here we aimed to uncover their contribution against *S. brasiliensis* in a murine model of disseminated sporotrichosis.

**Methods:**

Wild-type, Dectin-1 and/or Dectin-2 knockout, and IL-17A/F knockout C57BL/6J mice were challenged with *S. brasiliensis* in a model of systemic infection. Animals were monitored for parameters as survival and body weight loss. Immunological analyses as assessment of cytokines and immune cell profiling were conducted in the livers.

**Results:**

We showed that the receptors are essential for host survival, necessary to limit the fungal dissemination, and that their main effector functions can be related to shaping the T cell response, notably the cytotoxic CD8+ and Treg cell populations, instead of a conventional TH17 profile. While we also observed a contribution of IL-17 in the host defense, the cytokine is not involved in the restriction of the fungal growth.

**Discussion:**

Our results uncover dectin-1 and dectin-2 as novel determinants of protection against *S. brasiliensis*, but their effector function is not linked to the induction of IL-17 responses. Our fundings help to expand the understanding of the pathophysiology of this infection.

## Introduction

*Sporothrix brasiliensis* is an emerging fungal pathogen associated with epidemic outbreaks of a more aggressive form of the mycosis sporotrichosis (1). Belonging to the *Sporothrix schenckii* complex, *Sporothrix* spp. have always caused concern among the scientific community (2). However, the higher severity of *S. brasiliensis* infections linked to its strong epidemic potential has put this pathogen in the spotlight in recent years (3). Currently, *S. brasiliensis* has surpassed the other members of the *S. schenckii* complex as the main causative agent of sporotrichosis in Brazil, and unfortunately, it is spreading across several countries in Latin America (4), with isolated cases already being reported in Europe and North America (5).

Infections by *Sporothrix* spp. are primarily associated with contact with contaminated soil, plants, or organic matter (hence, the alias as the “gardener’s disease”) (6); however, for *S. brasiliensis*, zoonotic sporotrichosis and animal-to-human transmissions have been recognized as the main sources of contamination (1, 4). For instance, the ability of *S. brasiliensis* to infect animals, particularly stray cats, helps to explain its rapid dissemination and poses a great challenge for control by public health measures (4, 7). In addition, despite the subcutaneous nature of the mycosis, atypical (extra-cutaneous) presentations with invasive commitment linked to *S. brasiliensis* are more common and are on the rise (7, 8).

The higher virulence of *S. brasiliensis* is not fully understood and cannot be traced to a single trait. The most important features include: i) higher thermotolerance and thermodimorphic behavior (whereas the mycelial form is considered saprophytic and the yeast phase parasitic) (7)—but for *S. brasiliensis*, the zoonotic transmission occurs directly by yeast inoculation; ii) the ability to form biofilms (9); and iii) the production of enzymes, adhesion molecules, and melanin (10). Nevertheless, the interaction of these attributes with the host system is what determines the infection outcome.

From the host’s perspective, an even larger gap exists about the immune response triggered against *S. brasiliensis*, and only in recent years have some advances in addressing those questions been observed. In this context, innate immunity is our first layer of defense, and its operation requires pathogen detection mediated by pattern recognition receptors (11). The prototypical innate molecules Toll-like receptor 2 (TLR2) and TLR4 have been shown to be important for host defense in murine models of *S. brasiliensis* infection, mainly by regulating the effector function of phagocytes and the inflammatory response, although their deficiency did not compromise animal survival upon the fungal challenge (12, 13). The complement protein C3 and the surface molecule CD11b have also been shown to be important for the interaction between *S. brasiliensis* and macrophages (14).

The main sensors involved in fungal recognition, however, belong to the family of the C-type lectin receptors (CLRs). Dectin-1 (*CLEC7A* in humans and *Clec7a* in mice) and dectin-2 (*CLEC6A/Clec4n*) are the most studied CLRs, and we and others have reported their roles in the defense against a plethora of fungal pathogens (15–20). The induction of T helper 17 (T_H_17) responses is considered their canonical, but not solely, effector function (21).

Independent groups have suggested a marginal contribution of dectin-1 in the interaction between *S. brasiliensis* and phagocytes (22, 23), but these findings are limited to *in vitro* settings. Thus, the *in vivo* relevance of CLRs in anti-*S. brasiliensis* response is still an open question. Here, we proposed to evaluate the relevance of dectin-1/dectin-2 in the host response to *S. brasiliensis* in an experimental model of disseminated disease. We observed that the lack of these receptors severely compromised the ability to resist the fungal challenge. Curiously, the defective antifungal response in the absence of CLRs was not directly linked to an interleukin 17 (IL-17) response, but rather to a dysbalanced profile of cytotoxic CD8^+^ T cells and regulatory T cells (Tregs), which could be the result of a poor ability to activate dendritic cells. Our findings underscore dectin-1/dectin-2 as key determinants for an efficient host defense against *S. brasiliensis*.

## Materials and methods

### Mice

Female mice in C57BL/6J genetic background deficient for dectin-1 (*Clec7a* ^–/–^), dectin-2 (*Clec4n* ^–/–^), dectin-1/dectin-2 (*Clec7a* ^–/–^–*Clec4n* ^–/–^), IL-17A/IL-17F (I*l17a* ^–/–^–*Il17f* ^–/–^), and rag2 (*Rag2* ^–/–^) were used in this study (15). C57BL/6J wild-type (WT) mice were acquired from CLEA Japan (Tokyo, Japan) and co-housed with the knockout animals for at least 1 week prior to the experiments. All mice were maintained under specific pathogen-free conditions with a gamma ray-sterilized diet and acidified tap water (0.002 N HCl) *ad libitum*.

All experiments were conducted following the “Fundamental Guidelines for Proper Conduct of Animal Experiments and Related Activities in Academic Research Institutions under the Jurisdiction of the Ministry of Education, Culture, Sports, Science, and Technology” (Ministry of Education, Culture, Sports, Science and Technology, Japan, 2006). The Institutional Animal Care and Use Committee from Chiba University approved the protocols reported in this paper under process number A7-198.

### Fungal strain and inoculum preparation

The reference strain *S. brasiliensis* 5110 (*Sporothrix brasiliensis* Marimon MYA-4823; American Type Culture Collection, Manassas, VA, USA) was used throughout this study. The fungus was maintained in brain heart infusion (BHI) agar (BD, Franklin Lakes, NJ, USA) at 37°C with biweekly subcultures.

For inoculum preparation, the fungus was seeded in BHI agar plates and incubated for 5 days at 37°C. Colonies were harvested and washed in 0.1% Tween-80/PBS (phosphate-buffered saline) solution, resuspended in saline solution (0.9% NaCl), and kept at 4°C until use.

### *In vivo* infections

For *in vivo* infections, the animals were inoculated through the intravenous route (lateral caudal vein) with 5 × 10^6^ yeast cells in 100 μl of saline solution. Animal weight and survival were monitored daily for up to 27 days post-infection (dpi).

#### Fungal burden analysis

On the indicated dpi, the animals were euthanized by cervical dislocation, and the organs were perfused with ice-cold PBS before surgical removal. After being weighed, the organs were macerated in PBS through mesh sieves. Dilutions of the macerates were plated on PDA plates (Eiken Chemical, Tokyo, Japan), incubated at 30°C for 4 days, and the recovered colony-forming units (CFU) counted. Fungal burden was expressed as CFU per gram of organ. The macerates were centrifuged at 14,000 × *g* for 5 min, and the supernatants were collected and stored at −80°C for cytokine analysis (see below).

#### Histopathological analyses

On the indicated dpi, the livers were perfused with PBS and fixed overnight in commercial formalin solution (Fujifilm Wako, Osaka, Japan). Samples were embedded in paraffin, and sections were stained with routine hematoxylin–eosin, Grocott’s methenamine silver, or Masson’s trichrome staining method.

#### Isolation of liver leukocytes and flow cytometry analysis

Liver leukocytes were isolated from the sample macerates by Percoll centrifugation as described by Prosser et al. (24). The recovered cells were submitted to surface and intracellular staining for flow cytometry evaluation. For cell permeabilization, the commercial kits “Foxp3 Staining Buffer Set” (for CD4^+^ T-cell evaluation) and “Fixation & Permeabilization Buffer Set” (CD8^+^ T cells) (eBioscience, San Diego, CA, USA) were used according to the manufacturer’s instructions. Data were acquired with a FACSVerse flow cytometer (eight-color; BD, Franklin Lakes, NJ, USA) and analyzed using FlowJo (v.10.7.1 for Mac OS X; BD, Franklin Lakes, NJ, USA). The list of antibodies used for the analysis is provided in **Supplementary Table S1**, and representative gating strategies are shown in **Supplementary Figure S1**.

### Bone marrow-derived dendritic cells and *in vitro* infections

Bone marrow-derived dendritic cells (BMDCs) were generated from bone marrow cells harvested from the femur and tibia of the WT and *Clec7a* ^–/–^–*Clec4n* ^–/–^ mice by granulocyte–macrophage colony-stimulating factor (GM-CSF) differentiation protocol as previously described (15). On the day of the assay, 1 × 10^6^ BMDCs were stimulated with freshly harvested *S. brasiliensis* yeast cells (multiplicity of infection, 1:1) or 100 ng/ml of lipopolysaccharide (LPS) (from *Escherichia coli* O111:B4; Sigma-Aldrich, St. Louis, MO, USA) for 24h at 37°C and 5% CO_2_. The supernatants were harvested for cytokine measurements (see below), and the cells were stained with antibodies (**Supplementary Table S1**) for the flow cytometry analysis.

### Cytokine measurements

Cytokines [except for IL-10 and transforming growth factor beta (TGF-β)] were quantified using the BD Cytometric Bead Array assay according to the manufacturer’s instructions. Data were acquired using FACSVerse and analyzed with the FCAP Array software (v.3.0.1; BD, Franklin Lakes, NJ, USA). The detection limits were as follows: IL-1β = 1.9 pg/ml, IL-6 = 1.4 pg/ml, tumor necrosis factor (TNF) = 2.8 pg/ml, interferon gamma (IFN-γ) = 0.5 pg/ml, IL-4 = 0.3 pg/ml, IL-17A = 0.95 pg/ml, and IL-17F = 0.81 pg/ml.

The levels of IL-10 and TGF-β were quantified by sandwich ELISA using commercially available kits (DuoSet™ ELISA Development System, BioTechne/R&D Systems, Minneapolis, MN, USA) according to the manufacturer’s instructions. The adopted measurement range was 2,000–31.2 pg/ml.

### Statistical analysis

Statistical analyses were performed using the software GraphPad Prism (v.10 for OSX; GraphPad Inc., La Jolla, CA, USA). Data were screened for the detection of outliers using the ROUT method. The statistical test employed for each analysis, the sample size, and the number of replicates in each experiment are described in the figure legends. A *p-*value <0.05 was considered statistically significant.

## Results

### Dectin-1/dectin-2 are essential for protection against *S. brasiliensis* infection

**Figure 1:**
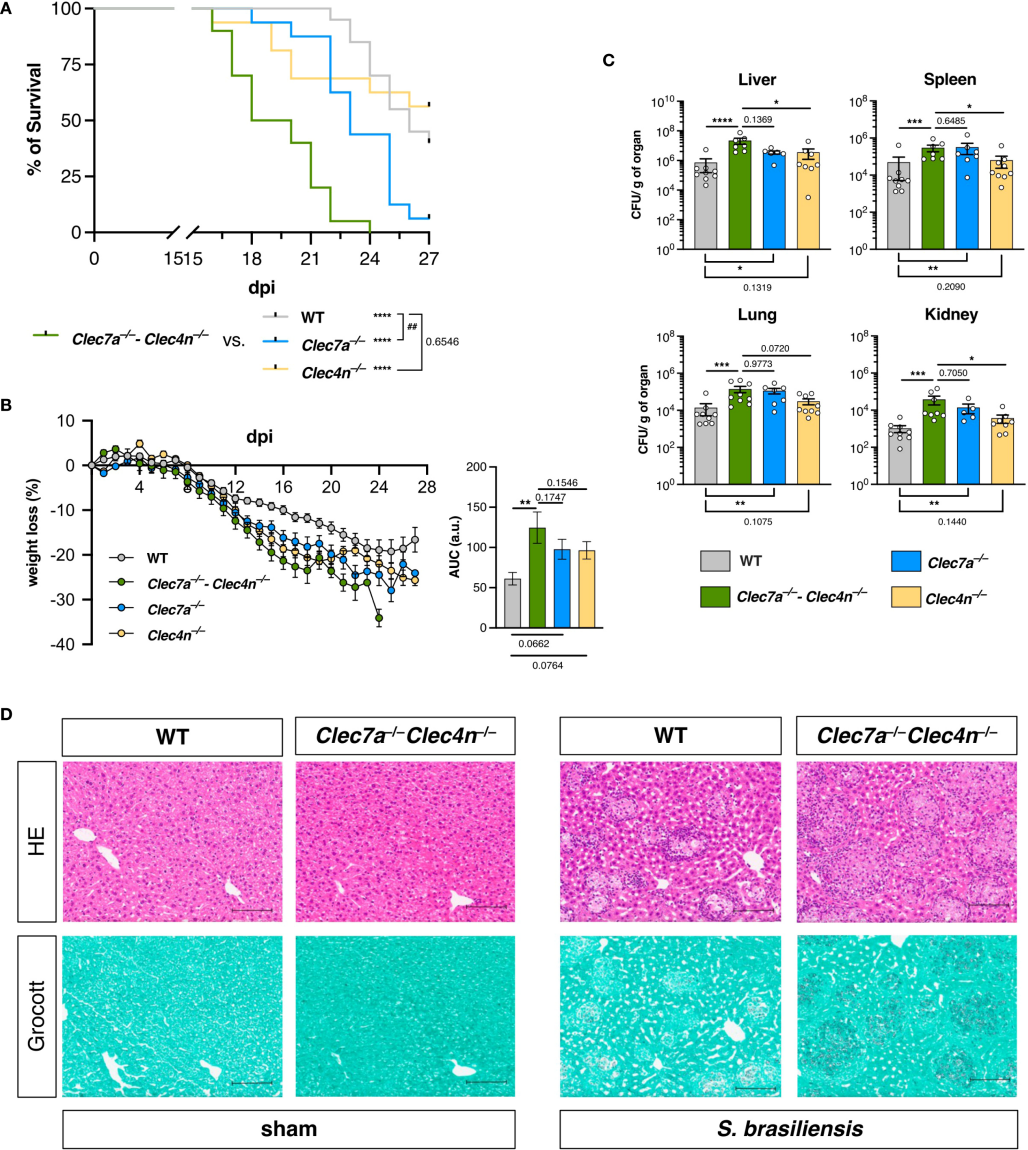
Dectin-1 and dectin-2 are essential for resistance against *Sporothrix brasiliensis* infection. **(A, B)** Wild type (WT), *Clec7a* ^−/−^, *Clec4n* ^−/−^, and *Clec7a* ^−/−^–*Clec4n* ^−/−^ mice were infected intravenously with 5 × 10^6^ yeast cells, and the survival **(A)** and body weight loss **(B)** were monitored for up to 27 days post-infection (dpi). *n* = 16–20 mice per group, pooled from two independent experiments. **(A)** Survival curves compared by log-rank (Mantel–Cox) test: **^##^** *p* < 0.01 (*vs*. WT); ****p < 0.0001 (*vs*. *Clec7a* ^−/−^ and *Clec4n* ^−/−^). **(B)** Body weight loss plots and area under the curve (AUC) bars shown as the mean ± SEM. One-way ANOVA and Fisher’s least significant difference (LSD) posttest: **p < 0.001. **(C)** Fungal burden in the organs harvested at 14 dpi. Data shown as colony-forming units (CFU) per gram of organ. *n* = 7–9 mice per group, pooled from two independent experiments. *Each dot* represents one mouse, and *bars* indicate the mean ± SEM. Kruskal–Wallis and Dunn’s posttest: *p < 0.05, **p < 0.01, ***p < 0.001, ****p < 0.0001. **(D)** Micrographs of the liver sections stained with hematoxylin–eosin (HE, *first row*) or Grocott’s methenamine silver (*second row*) methods. *Scale bars* represent 100 μm. Data representative of two (sham)–four (infected) mice per group from two independent experiments.

Systemic sporotrichosis is the most studied experimental model for investigating host–pathogen interactions and the immune response (25). We established a model of disseminated disease by administering *S. brasiliensis* yeast cells through the intravenous route and analyzed the outcome of the infection among WT and dectin-1 and/or dectin-2 knockout mice (**Figure 1**).

In agreement with our initial expectations, animals lacking dectin-1/dectin-2 were remarkably susceptible to *S. brasiliensis* infection (**Figure 1**). In addition to the enhanced mortality observed in the *Clec7a* ^−/−^–*Clec4n* ^−/−^ animals (**Figure 1A**), the infection caused a more intense weight loss during the course of the experiment compared with their WT counterparts (**Figure 1B**), indicating a more aggressive disease in the absence of the receptors. In line with this, the knockouts also presented higher fungal burdens in the liver, spleen, lungs, and kidneys (measured at 14 dpi), pointing to a systemic inability to restrain the pathogen dissemination (**Figure 1C**).

Interestingly, when the individual contribution of each receptor was analyzed using single knockout animals, dectin-1 appeared to play the dominant role, while the lack of dectin-2 alone did not remarkably alter the analyzed parameters (**Figure 1**). Nevertheless, it must be highlighted that the *Clec7a* ^−/−^ mice could not fully recapitulate the double-knockout profile, as the *Clec7a* ^−/−^–*Clec4n* ^−/−^ animals remained the most sensitive group. This suggests that dectin-2 is still involved in the protective response, but it may act by potentiating the functionality of dectin-1. Thus, to better characterize the host mechanisms involved, we followed the subsequent analyses with double-knockout animals.

These initial results indicate that dectin-1/dectin-2 are essential players in the host defense against *S. brasiliensis*, required for fungal restriction and maintenance of the host fitness.

#### Lack of dectin-1/dectin-2 does not enhance tissue inflammation

**Figure 2:**
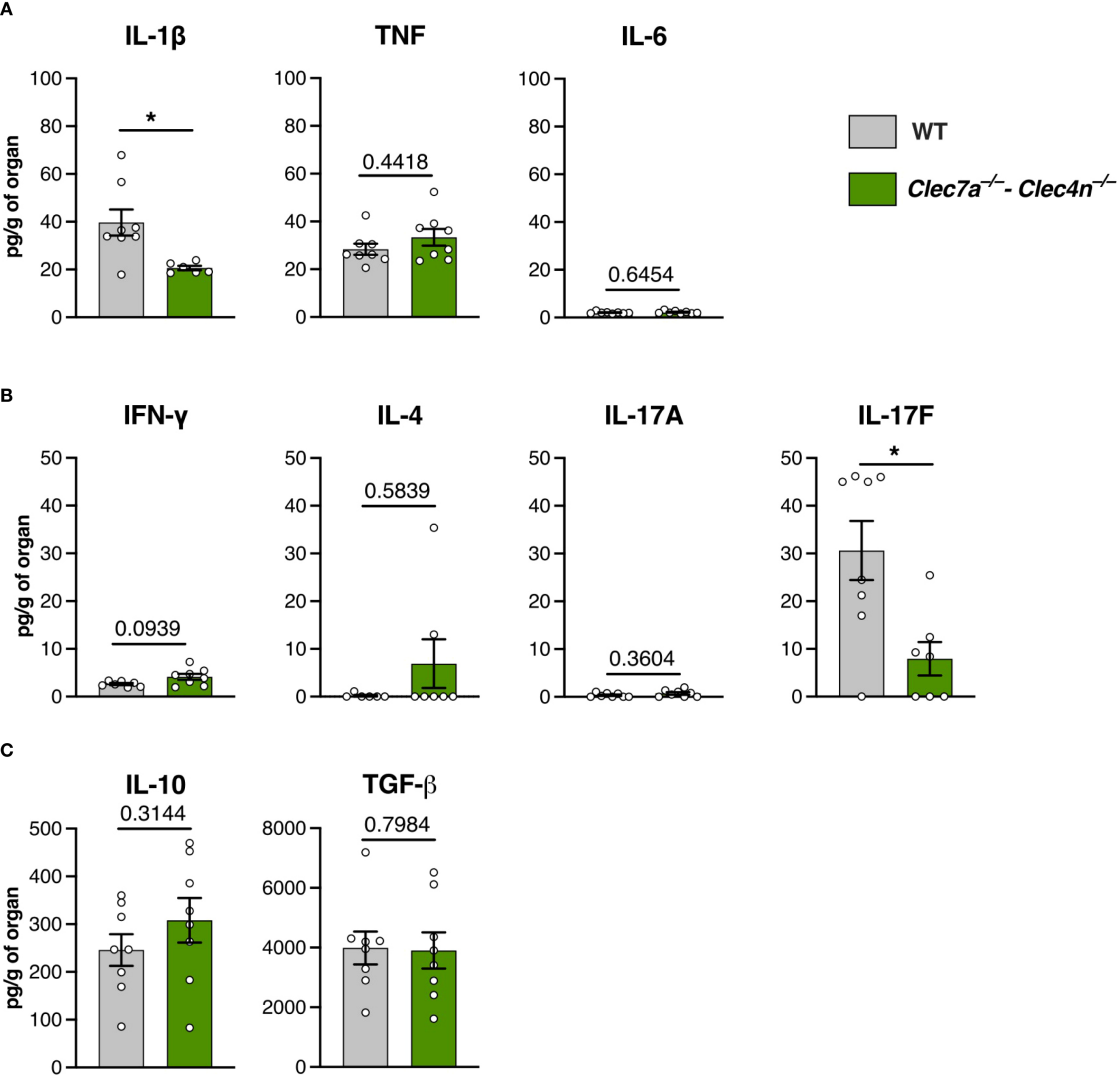
Cytokine profile in the liver macerates of *Sporothrix brasiliensis-*infected mice. Wild-type (WT) and *Clec7a* ^−/−^–*Clec4n* ^−/−^ mice were infected intravenously with 5 × 10^6^ yeast cells, and livers were harvested at 14 days post-infection (dpi). **(A)** Levels of IL-1β, TNF, and IL-6. **(B)** Levels of IFN-γ, IL-4, IL-17A, and IL-17F. **(C)** Levels of IL-10 and TGF-β. Data shown as picograms of cytokine per gram of organ. *n* = 8 mice per group, pooled from two independent experiments. *Each dot* represents one mouse, and *bars* indicate the mean ± SEM. Mann–Whitney *U* test: *p < 0.05.

The results from the fungal burden analysis pointed to the liver as the most compromised organ (**Figure 1C**), and it was chosen as a proxy for the response characterization. Initially, we confirmed the fungal colonization in the livers by histopathological analysis (**Figure 1D**). Interestingly, *S. brasiliensis* infection led to the development of diffuse, granuloma-like inflammatory foci around the fungal structures both in the WTs and the knockouts (**Figure 1D**, top row). However, as expected, in the *Clec7a* ^−/−^–*Clec4n* ^−/−^ mice, a massive fungal burden could be detected, as observed in the silver staining images (**Figure 1D**, bottom row). Thus, we next measured the levels of the cytokines classically involved in the inflammatory response and host defense to fungal pathogens (26) (**Figure 2**).

Curiously, despite the massive fungal colonization, the inflammatory cytokines were not proportionally augmented (**Figure 2A**). While TNF and IL-6 were not altered by the lack of dectin-1/dectin-2, lower levels of IL-1β were found in the knockouts. Interestingly, with regard to the cytokines associated with adaptive immunity, we found a predominance of the IL-17 response, particularly IL-17F, whose levels were compromised by the deficiency of the receptors (**Figure 2B**). Concurrently, we did not detect differences in the levels of the anti-inflammatory cytokines, i.e., IL-10 and TGF-β (**Figure 2C**).

Thus, instead of an overt inflammation driven by an unrestrained fungal growth, the lower levels of IL-1β and IL-17F would argue in favor of a hypothesis of dectin-1/dectin-2 promoting protection against *S. brasiliensis* through the induction of a prototypical type 3 (IL-17–driven) response, which is the paradigmatic branch of the adaptive immunity linked to resistance against fungal infections (27).

#### IL-17A/F are required for protection, but they do not regulate the fungal containment

**Figure 3:**
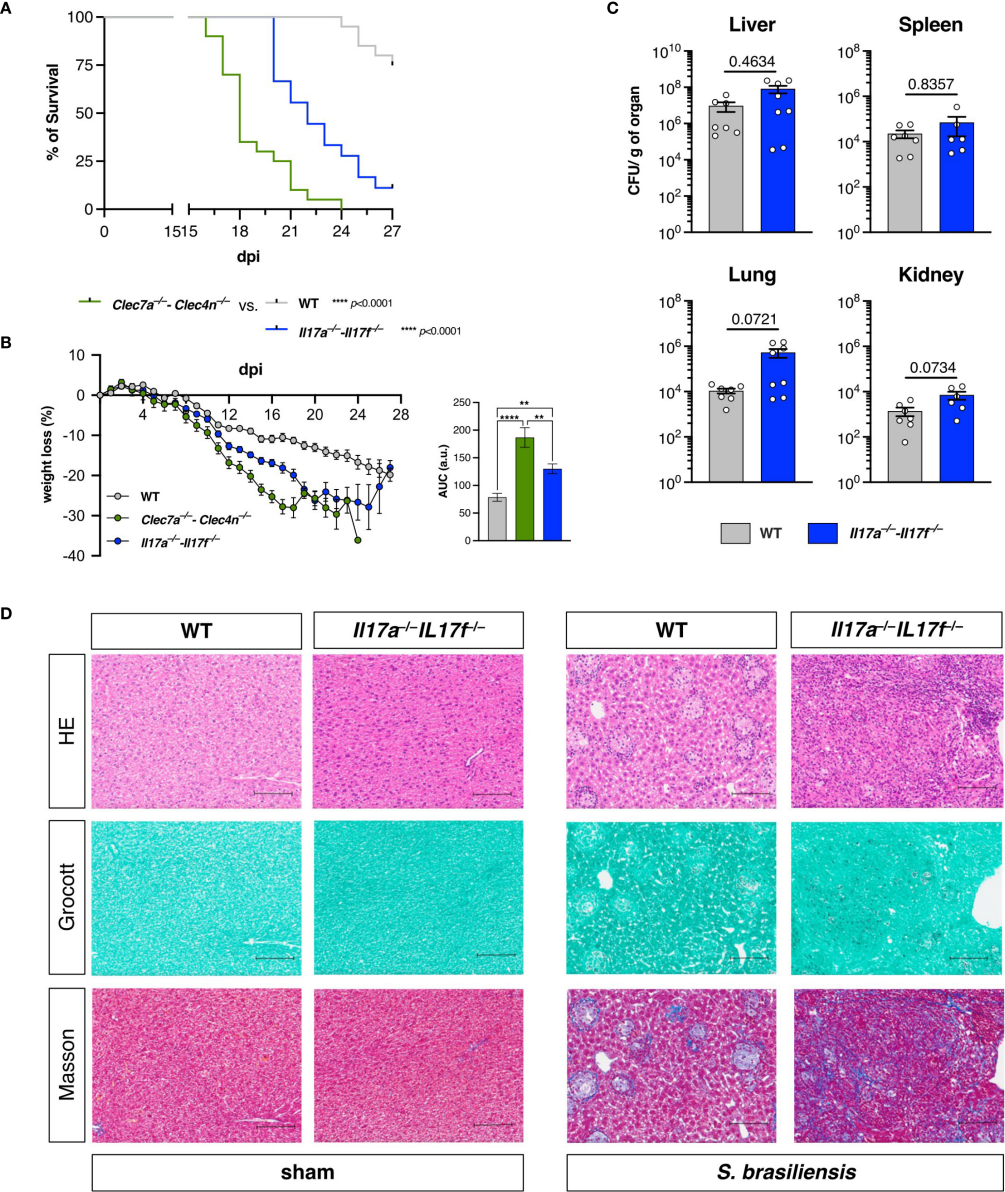
IL-17A/F promote resistance against *Sporothrix brasiliensis* infection, but do not alter fungal restriction. **(A, B)** Wild-type (WT), *Clec7a* ^−/−^–*Clec4n* ^−/−^, and *Il17a* ^−/−^–*Il17f* ^−/−^ mice were infected intravenously with 5 × 10^6^ yeast cells, and the survival **(A)** and body weight **(B)** loss were monitored for up to 27 days post-infection (dpi). *n* = 18–20 mice per group, pooled from two independent experiments. **(A)** Survival curves compared by log-rank (Mantel–Cox) test: ****p < 0.0001. **(B)** Body weight loss plots and area under the curve (AUC) bars shown as the mean ± SEM. One-way ANOVA and Fisher’s least significant difference (LSD) posttest: **p < 0.001, ****p < 0.0001. **(C)** Fungal burden in the organs harvested at 18 dpi. Data shown as colony-forming units (CFU) per gram of organ. *n* = 8 mice per group, pooled from two independent experiments. *Each dot* represents one mouse, and *bars* indicate the mean ± SEM. Mann–Whitney *U* test: no significance detected. **(D)** Micrographs of the liver sections stained with hematoxylin–eosin (HE, *first row*), Grocott’s methenamine silver (*second row*), or Masson’s trichrome (*third row*) method. *Scale bars* represent 100 μm. Data representative of two (sham)–four (infected) mice per group from two independent experiments.

To validate the importance of IL-17 in our model, we challenged the *Il17a* ^−/−^–*Il17f* ^−/−^ mice with *S. brasiliensis* and compared their infection outcome to those of the WT and *Clec7a* ^−/−^–*Clec4n* ^−/−^ groups (**Figures 3A, B**). As expected, IL-17 deficiency did compromise the host defense, leading to higher mortality and weight loss compared with the WT. Nonetheless, the *Clec7a* ^−/−^–*Clec4n* ^−/−^ mice were still more susceptible than their *Il17a* ^−/−^–*Il17f* ^−/−^ counterparts, indicating the involvement of additional mechanisms.

Astoundingly, despite the higher susceptibility of the IL-17 knockouts to the fungal challenge, the absence of the cytokines did not affect the fungal burden in any of the assessed organs (**Figure 3C**) as observed for dectin-1/dectin-2 deficiency (**Figure 1C**). Rather than uncontrolled fungal dissemination, the *Il17a* ^−/−^–*Il17f* ^−/−^ mice were colonized to the same levels as the WTs.

To obtain further insight into this observation, we also performed histopathological analysis of the livers from these animals (**Figure 3D**). Intriguingly, in contrast to the diffuse inflammatory foci observed, as shown in **Figure 1D**, deficiency in IL-17 led to a disorganized tissue structure, characterized by massive fibrosis, as revealed by Masson’s trichrome staining. It should also be noted that these features were not accompanied by widespread fungal growth, in agreement with the fungal burden data (**Figure 3C**), unlike what was observed in animals deficient in dectin-1/dectin-2 (**Figure 1**). These results suggest that the maintenance of host fitness against *S. brasiliensis* does not exclusively involve pathogen containment.

#### Dectin-1/dectin-2 do not shape the local T-helper cell profile

The disconnection between dectin-1/dectin-2 and IL-17 in the control of the fungal dissemination prompted us to re-evaluate whether type 3 immunity is the major response induced by the CLRs.

**Figure 4:**
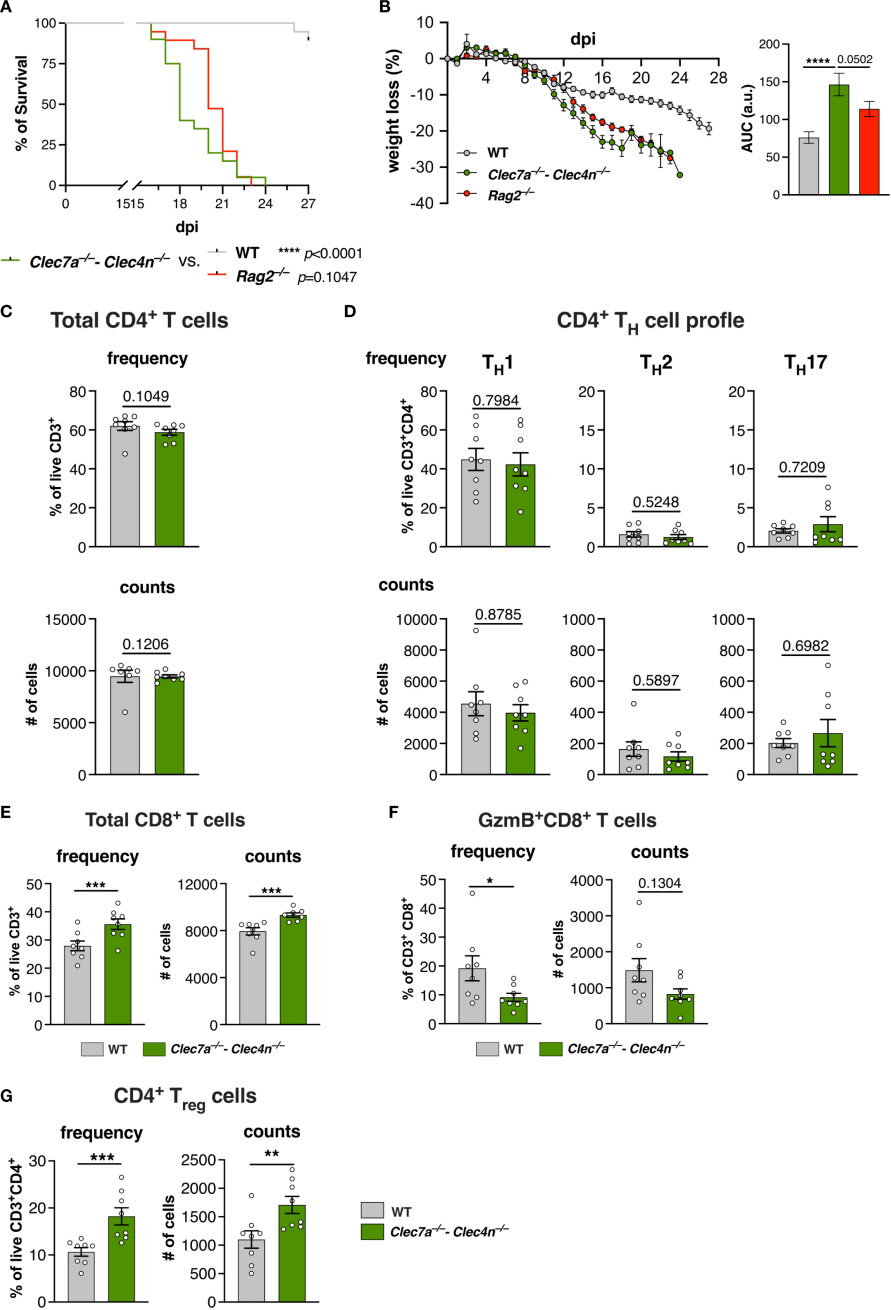
The T-cell profile is affected by the lack of dectin-1/dectin-2. **(A, B)** Wild type (WT), *Clec7a* ^−/−^–*Clec4n* ^−/−^, and *Rag2* ^−/−^ mice were infected intravenously with 5 × 10^6^ yeast cells, and the survival **(A)** and body weight loss **(B)** were monitored for up to 27 days post-infection (dpi). *n* = 19–20 mice per group, pooled from two independent experiments. **(A)** Survival curves compared by log-rank (Mantel–Cox) test: ****p < 0.0001. **(B)** Body weight loss plots and area under the curve (AUC) bars shown as the mean ± SEM. One-way ANOVA and Fisher’s least significant difference (LSD) posttest: ****p < 0.0001. **(C–G)** T-cell profile in the livers of infected mice harvested at 14 dpi. Frequency and counts of the total CD4 T cells **(C)**; T_H_1, T_H_2, and T_H_17 cells **(D)**; total CD8 T cells **(E)**; granzyme B (GzmB)-expressing CD8 T cells **(F)**; and regulatory T cells (Tregs) **(G)**. *n* = 8 mice per group, pooled from two independent experiments. *Each dot* represents one mouse, and *bars* indicate the mean ± SEM. Mann–Whitney *U* test: *p < 0.05, ***p < 0.001.

Initially, we aimed to confirm the requirement of lymphocytes for host resistance in our model by using animals knockout for Rag2 (15) and comparing their performance upon *S. brasiliensis* challenge (**Figures 4A, B**). Indeed, the lack of lymphocytes severely compromised the survival of the mice and led to a marked weight loss during the experiment. More importantly, their phenotype was virtually identical to *Clec7a* ^−/−^–*Clec4n* ^−/−^ mice, strongly implying that the lymphocyte response could be the primary effector function of dectin-1/dectin-2.

Subsequently, we aimed to identify the dominant adaptive response induced by *S. brasiliensis*. Firstly, we harvested splenocytes from infected mice, re-stimulated them with the pathogen yeast cells, and measured the hallmark cytokines in the culture supernatants (**Supplementary Figure S2**). Interestingly, IFN-γ was the predominant cytokine observed, whereas IL-4, IL-17A, and IL-17F were barely detected. Furthermore, the levels of IFN-γ were compromised by the lack of dectin-1/dectin-2. These results suggest that *S. brasiliensis* infection polarizes toward a type 1/T_H_1, not type 3/T_H_17, profile and that the process is instructed by dectin-1/dectin-2.

Thus, we next evaluated the profile of the CD4^+^ T-cell population in the livers of the infected animals (**Figures 4C, D**), which revealed no alterations in the population of total CD4^+^ T cells (**Figure 4C**). Furthermore, we characterized the subpopulations of T_H_ cells based on the expression of the classical transcription factors, i.e., T-bet (T_H_1), GATA3 (T_H_2), and RORγt (T_H_17) (28). In agreement with the splenocyte results (**Supplementary Figure S2**), the major subset was composed of T_H_1 cells, whereas the other subtypes were detected at lower levels (**Figure 4D**). Unexpectedly, no changes in their proportions were observed due to the lack of CLRs.

Therefore, even though dectin-1/dectin-2 might be needed to shape the T-cell response in secondary lymphoid organs, such as the spleen, this does not necessarily reflect in the cell profile at peripheral organs.

#### Deficiency of dectin-1/dectin-2 favors an immunosuppressed T-cell environment

The weak influence of dectin-1/dectin-2 over the T_H_-cell population prompted us to investigate other branches of the T-cell response, particularly CD8^+^ T cells and Tregs (**Figures 4E–G**).

Curiously, there was a pronounced influx of CD8^+^ T cells in the knockout group (**Figure 4E**). However, these cells showed lower levels of granzyme B (GzmB) compared with their WT counterparts (**Figure 4F**). Therefore, despite the higher presence of CD8^+^ lymphocytes, they displayed a dampened cytotoxic profile in the absence of dectin-1/dectin-2.

Remarkably, we could also detect a significant population of Tregs that was further increased in the *Clec7a* ^−/−^−*Clec4n* ^−/−^ mice (**Figure 4G**). Together with the impaired presence of cytotoxic CD8^+^ T cells, these results indicate that the lack of dectin-1/dectin-2 favors an immunosuppressed environment that might be less able to counter the fungal growth.

#### *S. brasiliensis* is a poor activator of dendritic cells

Our results indicate that dectin-1/dectin-2 are required for the balance of the lymphocyte response against *S. brasiliensis*. However, notwithstanding the profile of T cells that the receptors might enforce, rather than working directly on lymphocytes, CLRs act by shaping the profile of antigen-presenting cells, particularly dendritic cells (28).

**Figure 5:**
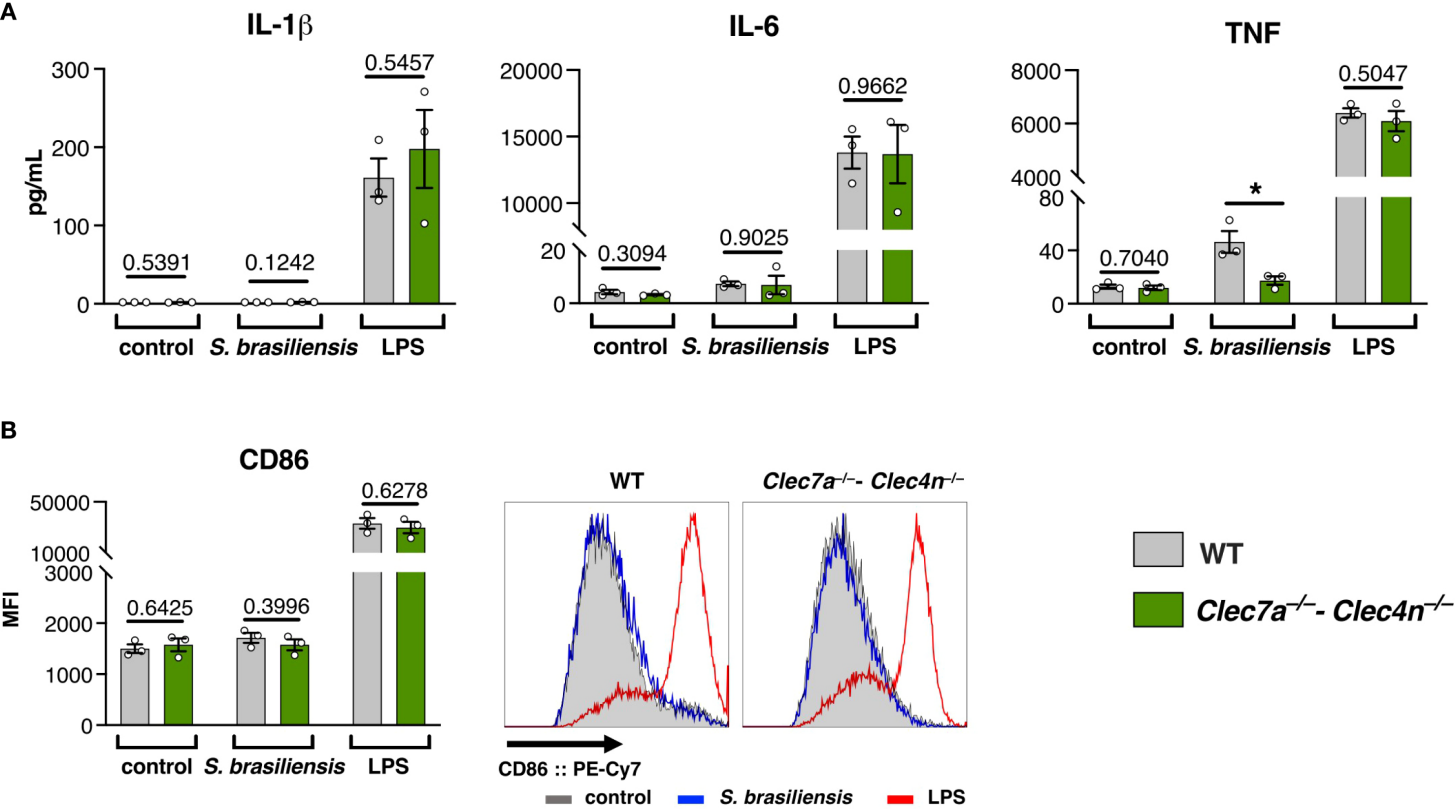
*Sporothrix brasiliensis* is a poor activator of bone marrow-derived dendritic cells (BMDCs). BMDCs were stimulated with *S. brasiliensis* or lipopolysaccharides (LPS) for 24h, and the activation markers were analyzed. **(A)** Levels of IL-1β, IL-6, and TNF in the culture supernatants. **(B)** Expression of CD86 on the BMDC surface (representative histograms on the *right side*). Data shown as the mean ± SEM, pooled from three independent experiments. Unpaired *t*-test: *p < 0.05.

To conciliate our findings, we analyzed the response of BMDCs stimulated with *S. brasiliensis* (**Figure 5**). Interestingly, we observed that the pathogen is a very weak BMDC activator. In contrast to the positive control LPS, *S. brasiliensis* triggered almost no cytokine production (**Figure 5A**) or expression of the co-stimulatory molecule CD86 (**Figure 5B**). We only observed a dectin-1/dectin-2-dependent production of TNF (**Figure 5A**), suggesting that these CLRs are still needed for a minimal level of cell activation.

The poor response of BMDCs could be indicative of a polarization toward a tolerogenic profile (29), and dendritic cells lacking dectin-1/dectin-2 show an even less activated phenotype that might reflect in potentiation of Treg differentiation, leading to a poorer cytotoxic environment that favors fungal proliferation.

## Discussion

The world is witnessing a mounting rise in the cases of fungal infections in recent years, partially driven by the emergence of novel, more aggressive pathogens such as *S. brasiliensis*, *Candida auris* (30), and *Trichophyton indotineae* (31). The understanding of the immunology of these infections is an urgent requirement for counteractions. Here, we showed that dectin-1 and dectin-2 are key receptors for host resistance against *S. brasiliensis*, but the infection itself displays features of dampened inflammation, which can sustain the chronic evolution of the disease.

The poor activation of BMDCs argues in favor of this hypothesis. In agreement with our data, human dendritic cells and granulocytes were also shown to be less responsive to *S. brasiliensis* stimulation (22, 23), while human macrophages were more sensitive (23). Interestingly, most of the immunogenicity of *S. brasiliensis* is suggested to be carried by extracellular vesicles secreted by the fungus instead of the fungal cell per se (32, 33). Antigen masking could be a possible strategy to escaping host detection, as *S. brasiliensis* has been shown to have a thicker cell wall with less antigen exposure (34, 35). Along this line, while García-Carnero et al. reported low *S. brasiliensis*-driven cytokine responses by human peripheral blood mononuclear cells (PBMCs) (36), Kischkel et al. detected high responsiveness in equivalent PBMC samples (37); however, the latter employed heat-killed yeast cells instead of native cells as the former, and the heat treatment might have enhanced the immunogenicity of the material. Alternatively, interspecies variables have to be taken into consideration, as human and murine immune cells may display distinct recognition patterns, as observed for *Candida albicans* (38), which might affect the interpretation of the profiles and limit direct extrapolations.

Although the definitive contribution of each phagocyte type to host defense needs to be addressed in the future, the overall poor inflammatory potential of *S. brasiliensis in vivo* might in fact contribute to the aggressiveness of the infection as the host response is moved toward an environment highly permissive to fungal dissemination and persistence. In this scenario, dectin-1/dectin-2 act by limiting the polarization of Tregs and favoring the cytotoxic activity of CD8 T cells.

In parallel, the finding that IL-17 is involved in host survival, but not due to a presumed antifungal activity, was unexpected and intriguing. In addition to the well-known roles of IL-17 cytokines in driving inflammatory responses, the cytokines are also involved in tissue maintenance and repair (39). In support of this idea, our histopathological analysis showed that the IL-17 knockouts did not exhibit overwhelming fungal dissemination, as observed in dectin-1/dectin-2 knockouts, but presented compromised, fibrotic livers, which may have contributed to the demise of the animals. The role of IL-17 in organ fibrosis remains a matter of debate, as this cytokine can exert either anti- or profibrotic effects according to the context of the underlying disease (40). Interestingly, it has been reported that IL-17A neutralization reduced the extent of granuloma formation in a model of infection with the parasite *Schistosoma japonicum* (41), and a similar mechanism might be occurring in *S. brasiliensis* infection, where IL-17 helps to limit the spread of fungal colonization. Hence, it is tempting to speculate that the primary role of IL-17 here might be containment of the tissue damage linked to the infection rather than driving a direct antifungal response.

The lower levels of IL-17F found in the liver of the *Clec7a* ^−/−^–*Clec4n* ^−/−^ animals suggest that dectin-1/dectin-2 can also regulate the local production of the cytokine; however, this feature might be playing a coadjutant role. Our results also hint that the cytokine might not come from a conventional T_H_17 cell and that alternative sources could include the local population of γδ T cells or group 3 innate lymphoid cells (42). Nonetheless, considering the decoupling in the phenotypes between the dectin-1/dectin-2 and IL-17 knockouts for fungal restriction, assessment of the roles of these cytokines requires an independent evaluation beyond the scope of this manuscript.

In contrast to our results, Batista-Duharte et al. reported a mixed IFN-γ/IL-17 (T_H_1/T_H_17) profile in their infected WT mice (43). However, they based their interpretations on phorbol myristate acetate (PMA)/ionomycin-stimulated cells, while we employed *S. brasiliensis* yeast cells (antigen-specific stimulation), which could explain the discrepancy in the results. In addition, they did not employ immunodeficient animals or pharmacological blockers to confirm the relevance of these cells/cytokines in their model, hindering comparisons about functionality between their study and ours. Nonetheless, they observed that *S. brasiliensis* induced a weaker inflammatory response compared with *S. schenckii-*infected animals, which was associated with the induction of Tregs. The same authors have also shown that Tregs are actively repressing the clearance of *S. schenckii* (44), indicating that this might be a common denominator of *S. brasiliensis-*driven pathogenesis.

Finally, we acknowledge that we did not investigate the contribution of B cells and antibodies here. However, it also needs to be recognized that the relationship between humoral immunity and the pathogenesis of fungal infections in general is still a poorly explored territory. In the sporotrichosis field, the glycoprotein gp70 is well known as the main virulence factor and antigenic component of *Sporothrix* spp (45). Although anti-gp70 antibodies can ameliorate the infection severity (46), most of the studies have focused on their use as biomarkers for diagnosis (47) or vaccine targets for therapy (48) rather than on their role in the immunopathogenesis of the infection. Far more obscure is the connection between dectin-1/dectin-2 and antibody production; however, it is suggested that β-glucan-driven dectin-1 activation might help in the production of IgG1 antibodies by B cells (49). Nevertheless, the humoral immunity is a field worth exploring in future works.

In summary, we showed here that dectin-1 and dectin-2 are key determinants of host protection against *S. brasiliensis* infection. However, rather than shaping a classical T_H_17 response, they are involved in counterbalancing the immunosuppressed environment driven by the fungal pathogen. Our work paves the way for the exploration of these receptors and their associated signaling pathways as key targets to uncover new therapeutic strategies.

## Data Availability

The raw data supporting the conclusions of this article will be made available by the authors, without undue reservation.
