# Supplementary material for: Dectin-1 and dectin-2 drive protection against Sporothrix brasiliensis in experimental sporotrichosis
Source: Front Immunol. 2025 Sep 25;16:1668445. doi: 10.3389/fimmu.2025.1668445 (PMC12507893; doi:10.3389/fimmu.2025.1668445)
Supplement: Supplementary file 1 [file DataSheet1.docx]

Supplementary Text

**Materials and Methods**

**Splenocyte restimulation**

Animals were infected as described in the main text and the spleens were harvested at 14dpi. The organs were macerated in PBS through mesh sieves and red cells were removed by osmotic shock with hypotonic solution. A total of 2 x10^6^ splenocytes per well were plated in a 96-well plate and stimulated with 4 x10^6^ heat-inactivated (60°C for 1h) *S. brasiliensis* yeasts. After 24h, the supernatants were harvested for cytokine measurements as described in the main text.

**SupplementaRY Figures**

**Supplementary Figure 1. Representative gating strategies for the flow cytometry analyses.** (A) analysis of CD4^+^ T helper cell subsets. (B) analysis of CD8^+^ T cells. (C) analysis of the BMDCs.

**Supplementary Figure 2. Dectin-1/dectin-2 favor IFN-γ in splenocytes.** Splenocytes from infected mice were stimulated with *S. brasiliensis* and the produced cytokines (IFN-γ, IL-4, IL-17A and IL-17F) were measured. nd, not detected. *N*= 8 mice per group pooled from two independent experiments. Each dot represents splenocytes from one mouse and bars indicate mean ± SEM. Mann­–Whitney U test: * *p*<0.05.

**Supplementary TABLE**

**Supplementary Table 1. List of reagents and antibodies for immunophenotyping**. (in alphanumerical order according to the target name)

| Reagent / Antibody | Clone | Manufacturer / Brand |
| --- | --- | --- |
| CD3ε APC anti-mouse | 145-2C11 | Biolegend |
| CD4 APC/Cyanine7 anti-mouse | GK1.5 | Biolegend |
| CD8a PE/Cyanine7 anti-mouse | 53-6.7 | Biolegend |
| CD11b PE anti-mouse/human | M1/70 | Biolegend |
| CD11c APC anti-mouse/human | N418 | Biolegend |
| CD86 PE/Cyanine7 anti-mouse | GL-1 | Biolegend |
| FOXP3 Alexa Fluor® 488 anti-mouse | MF-14 | Biolegend |
| anti-GATA3 BV421™ | 16E10A23 | eBioscience |
| Granzyme B APC/Cyanine7 anti-human/mouse | QA16A02 | Biolegend |
| I-A/I-E APC/Cyanine7 anti-mouse | M5/114.15.2 | Biolegend |
| Propidium Iodide | N/A | Sigma |
| anti-Mo ROR gamma (t) PE | B2D | Biolegend |
| anti-T-bet PE/Cyanine7 | 4B10 | Biolegend |
| Zombie Aqua™ Fixable Viability Kit | N/A | Biolegend |
